# Case of a Wound of the Throat Successfully Treated

**Published:** 1785

**Authors:** 


### II. *Case of a wound of the throat successfully treated. By Mr.* Thomas Payne, *Surgeon in London. Communicated in a letter to Dr.* Simmons.

ON the 23d of September, 1784, at six o'clock in the morning, I was desired to visit E. D. aged thirty-five years. He had cut his throat with a razor, the joint of which he secured firmly by means of his garter. The wound, which was between five and six inches long, he had began to make on the left side and to a considerable depth, as he divided part of the mastoideus muscle on that side, and the inferior muscles of the thyroid cartilage; and had likewise cut through the membranous substance between the thyroid and cricoid cartilages. The wound was continued on the right side in the same direction as on the left, but was not so deep; so that its course formed nearly one third of a circle. His pulse was barely perceptible, as he had lost at least three quarts of blood; the air made a rattling noise in its passage out of the wound, between the thyroid and cricoid cartilages. At first he was averse to having any thing done to the wound, but at length I prevailed prevailed on him to let me drefs it with agglutinants, compreffes, bandage, &c. (the bleeding required no particular attention) and then put him in a proper fituation in bed.

I was of opinion his wound would prove mortal, but upon my fecond vifit at eleven o'clock, I found his pulfe amazingly recovered. He was now perfectly rational, and willing to have any thing done towards his recovery.

When I faw him in the evening, he preffed me much to give my opinion ingenuoufly refpecting the probability of his wound being cured. Of courfe I gave him a favourable one, and endeavoured to convince him at the fame time, how much depended on his ftrictly obferving my orders. On examination, the dreffings fully anfwering my intention, I did nothing more, except prefcribing an anodyne draught.

September 24, he had paffed a tolerably good night—in the evening he became feverifh—and on the 25th, at five in the morning, I was called up, as the nurfe imagined he was lofing blood: upon removing fome of the dreffings I found it was not blood, but the natural difcharge fucceeding a recent wound. In the evening I removed nearly all the dreffings, and applied mild digeftive ointment, fticking plaifter, &c. and ordered a fomentation to be applied previous to the dreffing in the morning.

September 26, he had had a tolerably good night, but was much difturbed in his mind this morning. As he was coftive, I prefcribed an opening mixture, but this not operating, he had a ftrong cathartic clyfter.

September 27, he had paffed a good night, and the wound went on as well as I could expect. I prefcribed a faline mixture, with decoction of bark.

September 28, he was going on very well; as alfo on the 29th and 30th.

October 1, I found he had difturbed the dreffings very much in the night.

October 2, his attendants told me they had not heard, as ufual, the noife of the wind paffing through the wound this day, but the patient himfelf faid, it ftill continued to make its way.

October 3, every thing went on well, and in the evening he informed me, that he had not felt or heard any wind pafs the wound this day. On the 4th, the digeftion being complete, I perceived a part of the thyroid cartilage bare, but but not denuded of its perichondrium; the wound looked well, and incarnation was beginning to take place.

He continued to mend daily, and on the 26th of October left town, and went fixty miles into the country to his friends. The wound at this time was reduced to the fize of a filver fourpence; and, on the 4th of December his brother informed me that his throat was well, and that he was much better refpecting his nervous affections.

The applications to the wound were firft agglutinants, comprefs, bandage, &c.; afterwards fomentations, digeftives, cerate, fticking plaifter, aqua camphorata, efcharotics and dry lint, as circumftances required; but for the firft three weeks I always ufed fticking plaifter to keep the lips of the wound as near together as I could, and was particularly attentive to keep the head (by means of bandage and pofition of the body) always in the fame fituation.

The medicines before recited, fuch as the anodyne draught, opening mixture, and cathartic clyfter, were repeated as occafion required; but the bark was continued till the laft day of my vifiting him.

His

His regimen for the firſt fortnight was milk and barley water; the third week I ordered the addition of cuſtard and pudding; and the fourth ſtewed meat with broth.

I ordered the nurſe, particularly at firſt, to give him food but once in three or four hours, if he did not particularly require it (always adminiſtering fluids out of a ſmall tea pot). This I did in order to keep the parts as much at reſt as poſſible, there being ſuch conſiderable motion in the thyroid cartilage upon deglutition, as evinced the great utility that muſt ariſe from ſtrictly obſerving this particular.

The favourable event of this caſe being made known to the profeſſion, I flatter myſelf may prove of uſe to ſociety. With that hope only, I have taken the liberty, Sir, of communicating it to you for publication; and I ſhall think myſelf amply repaid, if what I have done be approved of, and practiſed by others with the ſame happy effect.

*Brook-Street, Groſvenor-Square,*

